# Functional regeneration of tissue engineered skeletal muscle *in vitro* is dependent on the inclusion of basement membrane proteins

**DOI:** 10.1002/cm.21553

**Published:** 2019-08-19

**Authors:** Jacob W. Fleming, Andrew J. Capel, Rowan P. Rimington, Darren J. Player, Alexandra Stolzing, Mark P. Lewis

**Affiliations:** ^1^ School of Sport, Exercise and Health Sciences Loughborough University Loughborough United Kingdom; ^2^ Wolfson School of Mechanical, Electrical and Manufacturing Engineering Loughborough University Loughborough United Kingdom

**Keywords:** bioengineering, regeneration, skeletal muscle physiology, tissue engineering

## Abstract

Skeletal muscle has a high regenerative capacity, injuries trigger a regenerative program which restores tissue function to a level indistinguishable to the pre‐injury state. However, in some cases where significant trauma occurs, such as injuries seen in military populations, the regenerative process is overwhelmed and cannot restore full function. Limited clinical interventions exist which can be used to promote regeneration and prevent the formation of non‐regenerative defects following severe skeletal muscle trauma. Robust and reproducible techniques for modelling complex tissue responses are essential to promote the discovery of effective clinical interventions. Tissue engineering has been highlighted as an alternative method, allowing the generation of three‐dimensional *in vivo* like tissues without laboratory animals. Reducing the requirement for animal models promotes rapid screening of potential clinical interventions, as these models are more easily manipulated, genetically and pharmacologically, and reduce the associated cost and complexity, whilst increasing access to models for laboratories without animal facilities. In this study, an *in vitro* chemical injury using barium chloride is validated using the C2C12 myoblast cell line, and is shown to selectively remove multinucleated myotubes, whilst retaining a regenerative mononuclear cell population. Monolayer cultures showed limited regenerative capacity, with basement membrane supplementation or extended regenerative time incapable of improving the regenerative response. Conversely tissue engineered skeletal muscles, supplemented with basement membrane proteins, showed full functional regeneration, and a broader *in vivo* like inflammatory response. This work outlines a freely available and open access methodology to produce a cell line‐based tissue engineered model of skeletal muscle regeneration.

**Abbreviations**
DM: differentiation medium
ECM: extracellular matrix
GM: growth medium
MPC: myogenic precursor cell

## 1 INTRODUCTION

For the majority of individuals skeletal muscle injuries are regenerative, restoring function to a level indistinguishable from that seen before injury. The severity of muscle wounds seen in clinical settings is highly varied, ranging from minor strains to significant muscle traumas such as volumetric muscle loss. Typically, wounds caused by military trauma or severe sports injury, do not retain full regenerative capacity and complete recovery is not observed (Belmont, McCriskin, Sieg, Burks, & Schoenfeld, 2012; Dharm‐Datta & McLenaghan, 2013; Wheatley et al., 2015). In addition, individuals with genetic conditions, collectively termed myopathies, also experience a lack of regeneration following injury (Dimachkie & Barohn, 2014; Flanigan, 2014; Nigro & Piluso, 2015; Shieh, 2013). In such situations the capacity of muscle to heal is impaired, resulting in non‐regenerative healing and subsequent fibrosis (Mann et al., 2011; Mueller, Van Velthoven, Fukumoto, Cheung, & Rando, 2016; Olson & Soriano, 2009), adipogenesis (Rose, 2002; Uezumi, Fukada, Yamamoto, Takeda, & Tsuchida, 2010; Uezumi et al., 2011), and in extreme cases osteogenesis (Belmont et al., 2012; Wheatley et al., 2015).

Mature adult skeletal muscle consists of aligned myofibres, encased within a basement membrane and a predominantly type I collagen extracellular matrix (ECM). These multinucleated cells contain the contractile apparatus of skeletal muscle, with the force generated transmitted through the ECM to the tendons (Cooke, 2004; Szent‐Györgyi, 2004). Located between the membrane of these multinucleated cells (sarcolemma) and the basement membrane resides a population of stem cells, termed satellite cells, which are required for muscle hypertrophy and regeneration (Hurme & Kalimo, 1992; Kuang, Kuroda, Le Grand, & Rudnicki, 2007; Mauro, 1961; Moss & Leblond, 1971). Following injury, satellite cells activated by the post injury environment proliferate rapidly producing large numbers of progeny (Doumit, Cook, & Merkel, 1993; Haugk, Roeder, Garber, & Schelling, 1995; Hurme & Kalimo, 1992), which express early markers of myogenic commitment (Kuang et al., 2007). It is these progeny which commit to the myogenic lineage fusing to regenerate the damaged myofibres and return full functionality (Cornelison et al., 2004; Seale et al., 2000). A number of studies have shown the importance of inflammatory mediators on muscle progenitor proliferation and differentiation, and so the role of inflammation in regeneration is seen as an essential coordinating event following injury (Arnold et al., 2007; Lu et al., 2010; Segawa et al., 2008; Summan et al., 2006).

Currently, limited pharmacological interventions are available, in clinical practice, to increase regenerative repair within injured and/or diseased populations. As such, individuals that fall into these groups are left with skeletal muscle which never recovers full function, and in many cases can cause long term pain and disability. The processes governing skeletal muscle regeneration are highly complex, relying upon the coordinated effects of multiple cell types *in vivo*. To date, studies of muscle regeneration have relied upon the use of animal models. However, attempts to reduce the reliance upon animals models, as per the 3Rs directive (EU, 2010; Russell, Burch, & Hume, 1959), in addition to difficulties translating animal data to human physiology (Boldrin, Muntoni, & Morgan, 2010; Gerry & Leake, 2014; Shanks, Greek, & Greek, 2009), demonstrates a requirement for new approaches. Recreating complex biological processes, such as skeletal muscle regeneration in *in vitro* tissue culture systems, requires sophisticated models of tissues which go beyond simple monolayer cultures.

Examination of cellular responses typically relies upon monolayer cell culture methods; however, these monolayer culture methods lack the advanced tissue hierarchy seen *in vivo*. The lack of spatial organisation in monolayer models may limit the capacity of cellular models to undergo complex physiological events, such as muscle regeneration. Tissue engineering has been highlighted as an alternative method, allowing the generation of three‐dimensional *in vivo* like tissues without the need for laboratory animals. Reducing the requirement for animal models promotes rapid screening of potential clinical interventions, as these models are easily manipulated genetically and pharmacologically and therefore are associated with reduced cost and complexity.

A number of tissue engineered models of skeletal muscle have been published which are capable of producing a 3D construct that contains aligned myofibres (Agrawal, Aung, & Varghese, 2017; Gilbert‐honick et al., 2018; Huang, Dennis, Larkin, & Baar, 2005; Juhas, Engelmayr, Fontanella, Palmer, & Bursac, 2014; Langelaan et al., 2011; Madden, Juhas, Kraus, Truskey, & Bursac, 2015; Martin et al., 2013; Rao, Qian, Khodabukus, Ribar, & Bursac, 2018; Sakar et al., 2012; Sharples et al., 2012; Vandenburgh et al., 2009; Vandenburgh, 2010), and in some cases capable of producing force when electrically stimulated (Capel et al., 2019; Juhas et al., 2014; Madden et al., 2015; Martin et al., 2017; Rao et al., 2018). These models have been used for a range of applications including preclinical drug screening and investigations of basic biology. To date only a single model has shown any regenerative capacity. This study employed a fibrin/Matrigel® hydrogel system containing primary skeletal muscle myoblasts from rats (Juhas et al., 2014, 2018). The culture system employed is based upon a bespoke 3D culture mould and uses primary rat myogenic precursor cells (requiring the sacrifice of small laboratory animals), negating some of the advantages associated with using engineered tissues as models. Therefore, a system that employs a freely available and open source 3D printed mould, to allow other investigators to accurately and rapidly reproduce the system, and does not use any primary animal tissue, represents a significant advance to the tissue engineering field.

Here, we present a comparative analysis of injury and regeneration in both monolayer and tissue engineered 3D culture systems using the murine skeletal myoblast cell line C2C12. Morphological analysis, gene expression and functional output are used to assess the differences and similarities between monolayer and 3D model systems and identify a system which most closely mimics *in vivo* skeletal muscle regeneration.

## 2 METHODS

### 2.1 Culture of C2C12 skeletal muscle myoblasts

C2C12 skeletal muscle myoblasts (below passage 10) were expanded using growth medium (GM); composed of 79% Dulbecco's Modified Eagle Medium (DMEM, Fisher Scientific, UK), 20% foetal bovine serum (FBS, Pan Biotech, UK) and 1% Penicillin/Streptomycin (P/S, Fisher). Cells were cultured in T80 flasks (NuncTM, Fisher) and incubated in a 5% CO_2_ humidified atmosphere at 37 °C until 80% confluence was attained. GM was changed every 24 hr during the expansion of cells.

### 2.2 Monolayer (2D) culture

Cells were plated into six well plates containing 0.2% gelatin coated glass cover slips at a density of 1 × 10^4^ cells/cm^2^. To examine the effect of surface matrix, experimental plates and coverslips were coated with a 1.5 mg/mL Matrigel® solution as per manufacturer's instructions (Corning, UK). Myoblasts were cultured to confluence in GM before being cultured in low serum differentiation medium (DM); composed of 97% DMEM, 2% Horse Serum (HS, Sigma Aldrich, UK) and 1% P/S. The total culture period was standardised at 2 days GM, followed by a further 3 days DM.

### 2.3 3D tissue engineered constructs

Collagen constructs were generated using C2C12 myoblasts, as previously published (Capel et al., 2019). Type I collagen only hydrogels were formed by the addition of 85% vol/vol type I rat tail collagen (First Link, UK; dissolved in 0.1 M acetic acid, protein at 2.035 mg/mL), with 10% vol/vol of 10X minimal essential medium (MEM, Gibco, UK). This solution was neutralised by the addition of 5 M and then 1 M sodium hydroxide (NaOH) dropwise, until a colour change to cirrus pink was observed. Collagen/Matrigel® constructs were generated by the addition of 65% vol/vol type I rat tail collagen, with 10% vol/vol of 10X minimal essential medium. This solution was neutralised as above. This was followed by the addition of 20% vol/vol Matrigel® (Corning®, Germany). Myoblasts were added to the neutralised collagen or collagen/Matrigel® solution at a density of 4 × 10^6^ cells/mL in a 5% vol/vol GM solution, before being transferred to the pre‐sterilised biocompatible polylactic acid (PLA) 3D printed inserts (Rimington, Capel, Christie, & Lewis, 2017) to set for 10–15 min in an incubator. Collagen only gels were set in 500 μL inserts (Rimington et al., 2018a), whilst Matrigel®/Collagen gels were set in 50 μL inserts (Rimington et al., 2018b, 2018c). All moulds used in this manuscript are freely available to download at the following URL: https://figshare.com/projects/3D_Printed_Tissue_Engineering_Scaffolds/36494. GM was added for 4 days and changed daily, before being changed to DM, refreshed every 2 days, for a further 10 days in culture. Figure S5 contains a cross sectional image and macroscopic image of deformed hydrogels to illustrate the morphology and appearance of mature control constructs.

### 2.4 Barium chloride injury and regeneration

Barium chloride (BaCl_2_) was chosen as an injurious stimulus due to previous *in vivo* publications and its high water solubility, allowing easy and reproducible *in vitro* application (Hardy et al., 2016; Mueller et al., 2016). Once cultures had reached maturity, as defined above, they were exposed to chemical injury by BaCl_2_. Prior to inducing injury, fresh DM was added to all conditions. Precisely 50 μl/mL of 12% wt/wt BaCl_2_ solution was then added to the medium for injury culture conditions, followed by a 6 hr incubation to cause injury. Following injury, cultures were washed once with phosphate buffered saline (PBS) to remove residual BaCl_2_ containing media. Control (no injury) and 0 hr (0 hrs) time points were collected at the end of injury incubation. Injury was followed by a regenerative period, and in all experiments this regenerative period mimicked exactly the protocol used to generate mature cultures, allowing comparison of regenerated cultures to controls. End GM time points were collected when regenerating cultures were changed from GM to DM. End DM time points were collected at the end of the experimental protocol.

### 2.5 Fluorescence staining

Cells and 3D constructs were fixed using a 3.75% formaldehyde solution (Sigma). The actin cytoskeleton of cells was identified using rhodamine phalloidin (1:500, Fisher) and nuclei were stained using 4′,6‐diamidino‐2‐phenylindole (DAPI, 1:1,000, Fisher) in tris‐buffered saline (TBS) for 2 hr. Coverslips and 3D constructs were then washed (3 × 15 min, TBS). Coverslips were mounted directly onto microscope slides using Fluoromount™ mounting medium (Sigma). Constructs were transferred to polysine adhesion microscope slides (Fisher) and mounted under glass coverslips, again using Fluoromount™ mounting medium.

### 2.6 Image collection and analysis

Images were captured using a Leica DM2500 (monolayer) or a Zeiss LSM 880 confocal (3D) microscope. Morphological measures; fusion index (number of nuclei in myotubes represented as a percentage of the total number of nuclei in the image frame), myotube density per 100 μm (number of myotubes measured intersecting a line drawn perpendicular to the long axes of the construct, averaged from five points per image), percentage coverage (myotube width multiplied by myotubes per 100 μm) and average myotube width were all conducted manually. Total nuclei were calculated using an in‐house macro implemented in ImageJ (Schindelin et al., 2012). Analysis was conducted from nine monolayer images taken across three coverslips per biological repeat, or from a 21‐image tile scan of a 3D construct for every condition, derived from *n* ≥ 3 biological repeats.

### 2.7 Assessment of muscle function by electrical stimulation

Electric field stimulation was used to assess the functional capacity (force generation) of tissue engineered constructs. Constructs were washed twice in PBS, and one end of the construct removed from the supporting mould pin. The loose end of the construct was then attached to the force transducer (403A Aurora force transducer, Aurora Scientific, Canada) using the eyelet present in the construct. The construct was positioned to ensure its length was equal to that before removal from the pin and covered (3 mL) with Krebs‐Ringer‐HEPES buffer solution (KRH; 10 mM HEPES, 138 mM NaCl, 4.7 mM KCl, 1.25 mM CaCl_2,_ 1.25 mM MgSO_4_, 5 mM Glucose, 0.05% Bovine Serum Albumin in dH_2_O, Sigma). Aluminium wire electrodes, separated by 10 mm, were positioned either side of the construct to allow for electric field stimulation. Impulses were generated using LabVIEW software (National Instruments, Berkshire, UK) connected to a custom‐built amplifier. Maximal twitch force was determined using a single 3.6 V/mm, 1.2 ms impulse and maximal tetanic force was measured using a 1 s pulse train at 100 Hz at 3.6 V/mm, generated using LabVIEW 2012 software (National Instruments). Where possible, twitch and tetanus data were derived from three contractions per construct, and a minimum of two constructs per time point per biological repeat. Data was acquired using a Powerlab system (ver. 8/35) and associated software (Labchart 8, AD Instruments, UK).

### 2.8 RNA extraction and RT‐PCR

RNA was extracted using TRIReagent® chloroform extraction, according to manufacturer's instructions (Sigma). RNA concentration and purity were obtained by UV spectroscopy (Nanodrop 2000, Fisher). For snap frozen 3D constructs, TRIReagent® extraction was augmented by mechanical disruption of hydrogels via addition of metal beads in round bottomed Eppendorf's (Starlab, UK) in a TissueLyser II (Qiagen, UK) for 5 min at 20 Hz. Following disruption, beads were removed, and RNA was isolated as for monolayer samples.

All primers (Table 1) were validated for 5 ng of RNA per 10 μL real time‐polymerase chain reaction (RT‐PCR) reaction. RT‐PCR amplifications were carried out using Power SYBR Green RNA‐to‐CT 1 step kit (Qiagen, UK) on a 384 well ViiA Real‐Time PCR System (Applied Bio‐systems, Life Technologies), and analysed using ViiA 7RUO Software. RT‐PCR procedure was: 50 °C, 10 min (for cDNA synthesis), 95 °C, 5 min (reverse transcriptase inactivation), followed by 40 cycles of 95 °C, 10 s (denaturation), 60 °C, 30 s (annealing/extension). Melt analysis was then carried out using standard ViiA protocol. Relative gene expressions were calculated using the comparative CT (^ΔΔ^CT) method giving normalised expression ratios (Schmittgen & Livak, 2008). RPIIβ was the designated housekeeping gene in all RT‐PCR assays and no sample controls for each primer set were included on every plate.

**Table 1:** Forward and reverse primer sequences for all primers used for RT‐PCR expression analysis

Primer	Sequence (5′ to 3′)	Product length	NCBI reference sequence
RPIIβ	Fw‐GGTCAGAAGGGAACTTGTGGTAT	197	NM_153798.2
	Rv‐GCATCATTAAATGGAGTAGCGTC		
Myod	Fw‐CATTCCAACCCACAGAAC	125	NM_010866.2
	Rv‐GGCGATAGAAGCTCCATA		
Myog	Fw‐CCAACTGAGATTGTCTGTC	173	NM_031189.2
	Rv‐GGTGTTAGCCTTATGTGAAT		
Myh3	Fw‐CATATCAGAGTGAGGAGGAC	86	NM_001099635.1
	Rv‐CTTGTAGGACTTGACTTTCAC		
Tnf	Fw‐TCAACAACTACTCAGAAACAC	130	NM_013693.3
	Rv‐AGAACTCAGGAATGGACAT		
Il6	Fw‐AAGAAATGATGGATGCTACC	164	NM_001314054.1
	Rv‐GAGTTTCTGTATCTCTCTGAAG		
Mcp1	Fw‐CAAGATGATCCCAATGAGTAG	87	NM_011333.3
	Rv‐TTGGTGACAAAAACTACAGC		
Pparg	Fw‐AAAGACAACGGACAAATCAC	195	XM_017321456.1
	Rv‐GGGATATTTTTGGCATACTCTG		
Runx2	Fw‐ACAAGGACAGAGTCAGATTAC	197	XM_006523545.2
	Rv‐CAGTGTCATCATCTGAAATACG		

*Note*: Product length and NCBI gene reference determined using NCBI primer blast.

Abbreviation: RT‐PCR, real time‐polymerase chain reaction.

### 2.9 Statistical analysis

Statistical significance of data was determined using IBM© SPSS© Statistics version 23. Mauchly's test of sphericity and Shapiro–Wilk tests were used to confirm homogeneity of variance and normal distribution of data, respectively. Where parametric assumptions were met, an ANOVA test was used to identify significant interactions. Where significant interactions were observed, Bonferroni post‐hoc analyses were used to analyse differences between specific time‐points. Non‐parametric Kruskal–Wallis analysis was undertaken where data violated parametric assumptions. Mann–Whitney (*U*) tests were then used, with a bonferonni correction, to identify the differences between groups. All data is reported as mean ± *SD*. Significance was assumed at *p* ≤ .05.

## 3 RESULTS

### 3.1 Monolayer muscle cultures exhibit only a partial regenerative response following injury

To cause a wounding insult in 2D, a mechanism of injury is required that specifically removes myotubes, without causing excessive damage to the mononuclear cell population. The chemical BaCl_2_ was selected, demonstrating a dose dependent ability to specifically remove myotubes (Figure S1). Furthermore, BaC1_2_ has been documented to induce injury in animal models of skeletal muscle regeneration (Hardy et al., 2016).

**Figure 1:**
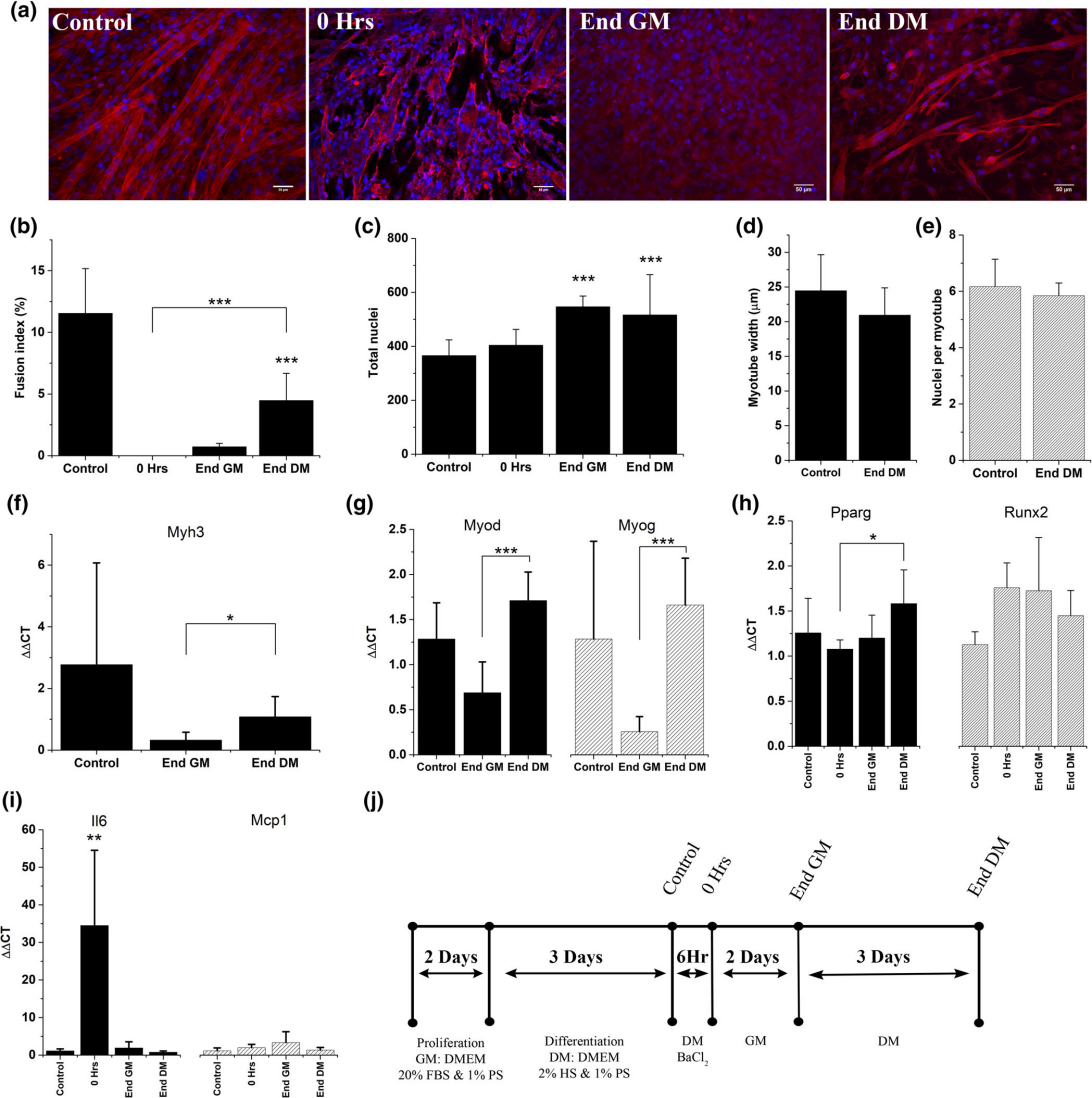
**Treatment of differentiated C2C12 cultures with BaCl_2_ specifically removes myotubes from culture and initiates a regenerative response**. (a) ×20 widefield micrographs of recovery time points in C2C12s. Stained for actin (phalloidin, red) and nuclei (DAPI, blue), scale bars represent 50 μm. (b) Fusion index, (c) total nuclei, (d,e) measures of myotube maturity. Values for 0 hr and 2 days post injury have been omitted as too few myotubes were present in these conditions to accurately measure these variables (b–e) Graphs express mean ± *SD*, asterisks above bars denotes significance from control, ***denotes significance *p* < .001, (f–i) RT‐PCR analysis of cellular developmental and inflammatory markers. All graphs display mean ± *SD*, *denotes significance *p* < .05, **denotes significance *p* < .01, ***denotes significance *p* < .001. (j) Experimental timeline for generation of differentiated C2C12 culture, injury and subsequent recovery. RT‐PCR, real time‐polymerase chain reaction

BaCl_2_ insult in monolayer cultures caused total removal of myotubes, demonstrated by the significant ablation of fused nuclei (*p* < .001), however no reduction in total nuclei number indicates the retention of mononuclear cells following injury (Figure 1a–c). Following a further 2 days culture in GM, nuclei number was significantly increased (*p* < .001), without a corresponding increase in fusion index, outlining a proliferative response to BaCl_2_ insult without myoblast fusion. Following 3 days in DM fusion index recovered from 0 to 48% of the value at control (*p* < .001), but remained significantly reduced compared to control (*p* < .001). Nuclei per image frame remained significantly elevated compared to control after 5 days regeneration (*p* < .001), but returned to baseline following a further 3 days regeneration (Figure S2). No difference was observed in indicators of maturity; myotube width or nuclei per myotube, from control (Figure 1d,e, *p* > .05).

Increases in nuclei number without commitment to fusion, suggests a blockade in myogenesis following BaCl_2_ insult. As such, an examination of genes involved in myogenesis and skeletal muscle maturity were undertaken. Embryonic myosin heavy chain, Myh3, expression correlated with fusion index (Figure 1f), although no significant reduction was identified between control and 5 days (*p* = .09). The transcriptional regulators of myogenesis, Myod and Myog, expression levels were supressed although not significantly 2 days post injury (Figure 1g, *p* > .05). Both Myod and Myog showed significant increases (*p* < .001) during DM phase of recovery, consistent with an activation of myogenesis and a level of regeneration. Non‐myogenic commitment to osteogenic lineage was measured via the expression of Runx2, although no significant increase above control was observed (*p* > .05). An upward trend in the expression of the adipogenic marker Pparg across recovery was evident, with significant increases in this gene observed after 5 days regeneration compared to immediately post injury (Figure 1h, *p* = .041). The role of inflammatory signalling in injury and regeneration is well established. As such, inflammatory genes encoding for interleukin‐6 (Il6) and macrophage chemoattractant protein‐1 (Mcp1/Ccl2) were used to analyse levels of inflammation induced by BaC1_2_ administration. Il6 expression increased 36‐fold (*p* = .0013) when compared to immediately post injury. This was, completely resolved following 2 days recovery in GM (*p* < .05). Mcp1 displayed no significant upregulation at any time‐point throughout the recovery process (Figure 1i).

The recovery of myogenic genes Myod and Myog (Figure 1g) suggested that although myogenic precursors were present in cultures after 5 days post injury, myogenic progression was not sufficiently advanced to enable the fusion required at this time‐point to fully regenerate ablated myotubes. To examine whether extended regenerative periods were sufficient to initiate full recovery, time in DM was extended by a further 3 days, taking the total regeneration time to 8 days. Despite this no increase in fusion index was observed between 5 and 8 days regeneration (Figure S2).

### 3.2 Type I collagen based tissue engineered muscles lack regenerative capacity following injury

A widely published system for the culture of tissue engineered skeletal muscle is the use of type I collagen hydrogels (Smith, Passey, Greensmith, Mudera, & Lewis, 2012; Vandenburgh et al., 2009), generating aligned myotubes via longitudinal tension. This well‐established model was utilised to examine if the incorporation of cells into a tissue engineered environment was sufficient to support full regeneration.

**Figure 2:**
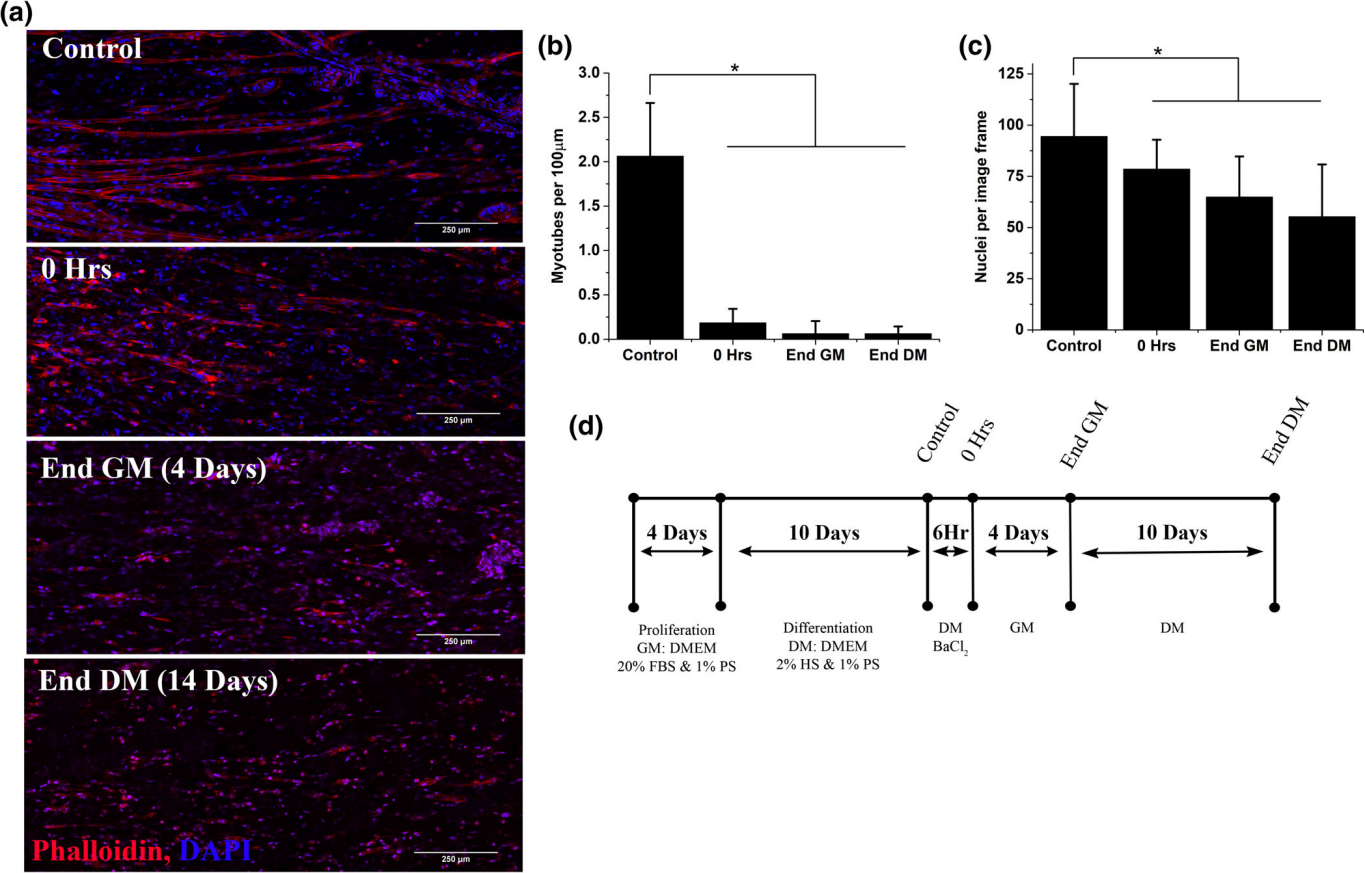
**Type I collagen hydrogels lack regenerative capacity following injury**. (a) representative confocal microscope tile scans at ×40 magnification consisting of 21 individual images. Stained with phalloidin (red) and DAPI (blue) to identify actin and nuclei, respectively. Scale bars denote 250 μm. (b) Myotube density expressed as myotubes per 100 μm. Mean ± *SD*. (c) Total nuclei per image frame. Mean ± *SD*, *p < .05. (d) Experimental time course of the 28 day 3D recovery experiment

Following insult, significant reductions in myotube density were observed (*p* < .001, Figure 2a,b), accompanied by decreased total nuclei (Figure 2c). During the regenerative period, recovery of myotube density was completely inhibited (Figure 2a), with reductions in nuclei number also evident across time (Figure 2c). This data suggests that hydrogels composed solely of type I collagen do not support regeneration following injury, and so are not a viable *ex vivo* model of skeletal muscle regeneration.

### 3.3 Inclusion of basement membrane proteins in the form of Matrigel® is sufficient to support regeneration in 3D

The potential lack of a stem cell like niche between the sarcolemma and basement membrane in hydrogels consisting of type I collagen only, could explain the inability to regenerate. To confirm the requirement for a basement membrane, hydrogels containing the basement membrane supplement Matrigel® were injured and allowed to regenerate. Hydrogels composed of collagen/Matrigel® were cultured in 50 μl inserts compared to 500 μl for collagen only hydrogels, to increase experimental throughput. The comparison of the mould sizes has been made previously and shown to be consistent (Capel et al., 2019).

**Figure 3:**
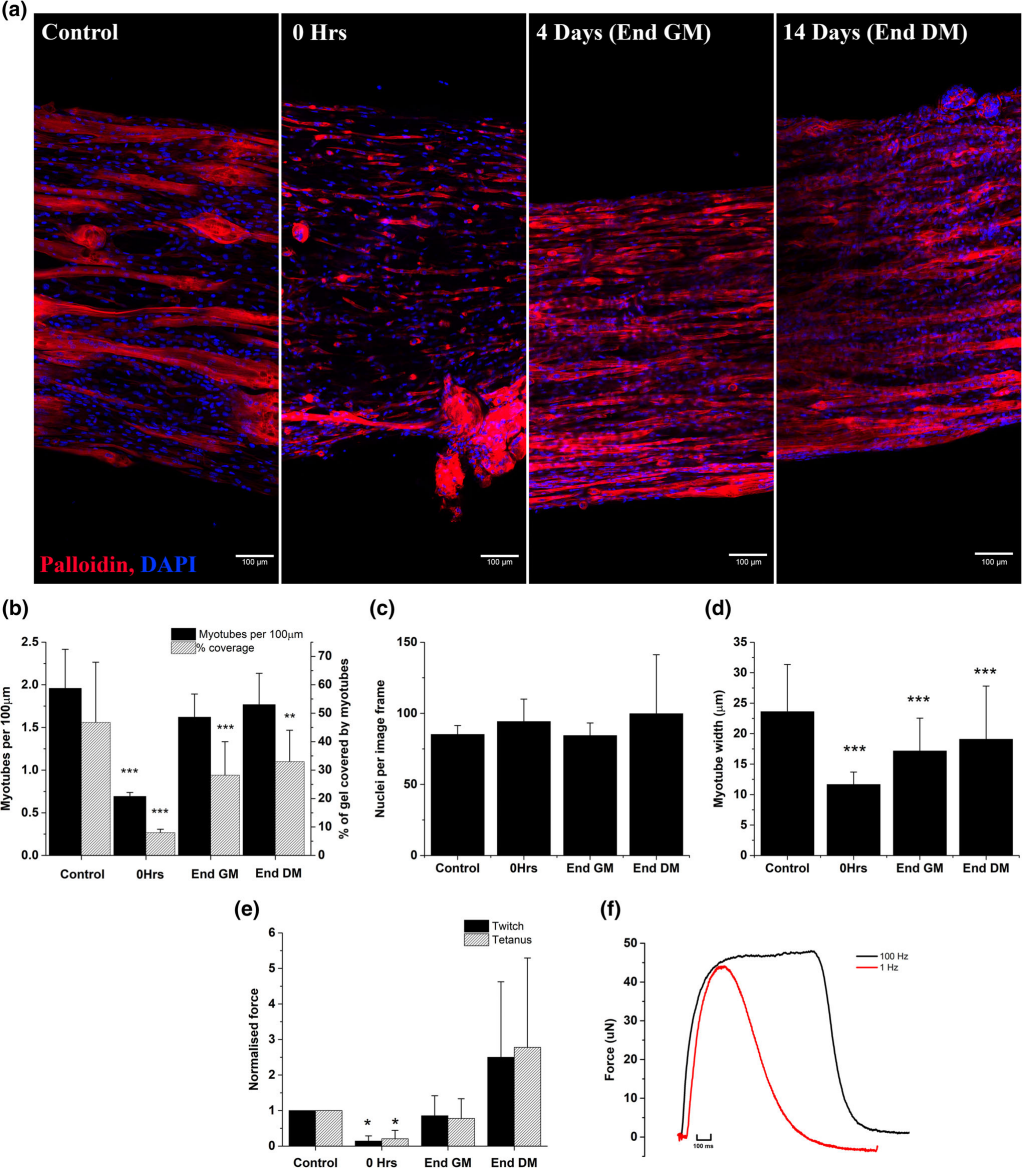
**Inclusion of Matrigel® allows 3D culture models to support regeneration following insult from BaCl_2._** (a) Representative tile scans at ×40 magnification, phalloidin staining (red) and DAPI (blue) identify actin and nuclei, respectively. Scale bars represent 100 μm. (b) Myotube density expressed as myotubes per 100 μm (solid bars) and percentage of gel occupied by myotubes (hashed bars). Mean ± *SD*, significance from control denoted by asterisks above bars, **p < .01, ***p < .001. (c) Nuclei per image frame. Mean ± *SD*. (d) Myotube width (μm). Mean ± *SD*, significance from control ***p < .001. (e) Force output data, twitch (1 Hz, solid bars) and fused tetanus (100 Hz, hashed bars) peak force expressed as mean ± *SD*, significance from control, **p < .01. (f) Representative force traces for C2C12 constructs stimulated at different frequencies to achieve; single twitch (1 Hz) and fused tetanus (100 Hz)

Immediately following injury in collagen/Matrigel® hydrogels, myotube density and coverage were significantly reduced (*p* < .001, Figure 3a,b), demonstrating that 6 hr BaCl_2_ exposure remains sufficient to induce injury. Following 4 days (End GM) of regeneration, myotube density recovered to control levels and remained stable over the remaining 10 days (End DM, Figure 3b). Myotube coverage remained significantly reduced at both End GM and End DM time points (*p* < .001, *p* = .015, Figure 3b). This is due to significant reductions in myotube width from 23.6 μm (control) to 19.1 μm at 14 days following injury (Figure 3d). No variation over time was seen in total nuclei per image frame, and so no proliferative response was observed (Figure 3c). Morphological decreases in myotube width did not translate to reduced functional output (Figure 3e), with force reduced following injury, but recovering completely at End GM time point. Although not statistically significant, force generation was enhanced at End DM. Twitch force outputs were on average 2.5‐fold higher and tetanus 2.8‐fold higher than control. Figure 3f shows representative force traces at two frequencies for control gels, no difference in force trace shape was observed during recovery.

**Figure 4:**
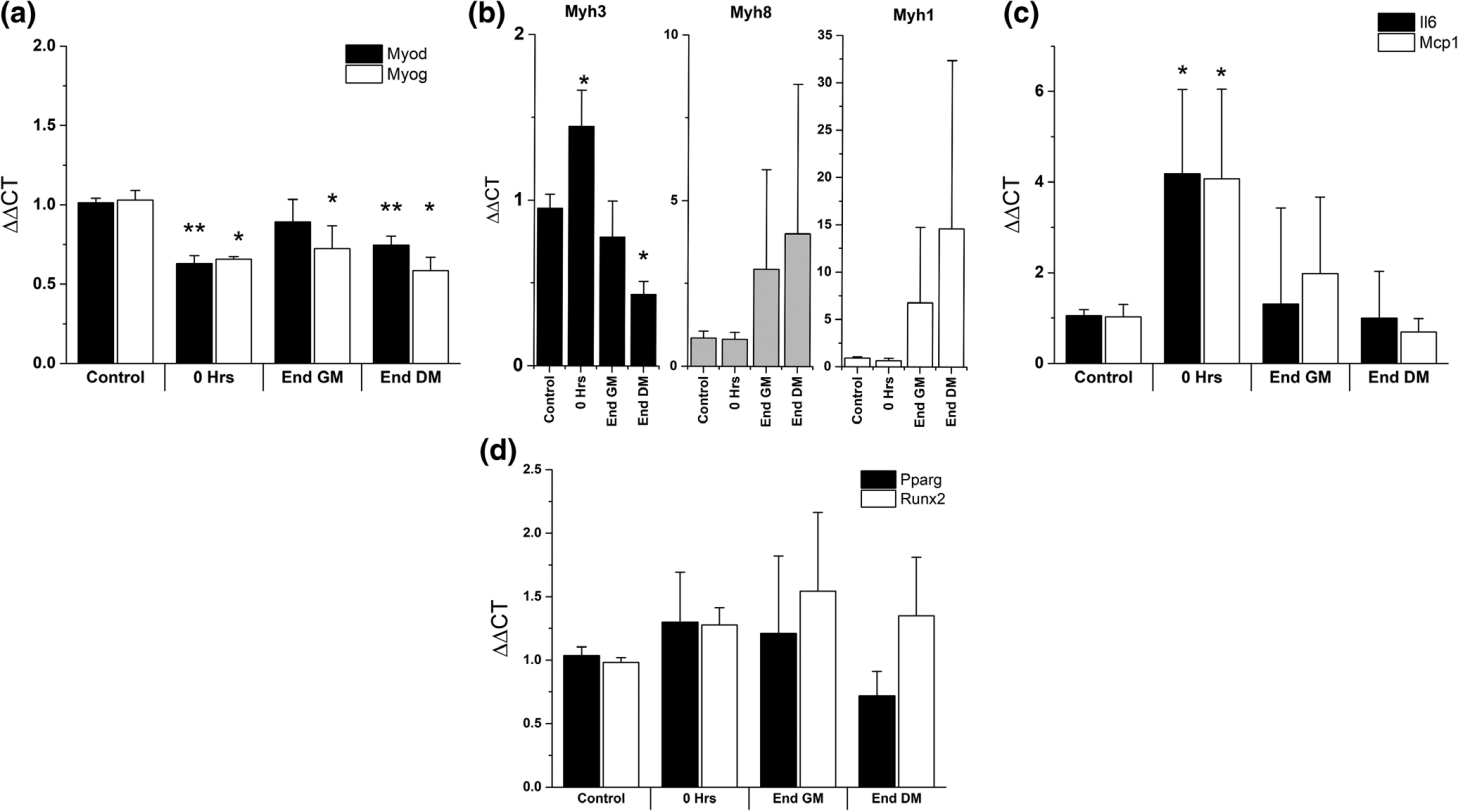
**RT‐PCR analysis of gene expression during recovery of 3D tissue engineered muscle**. (a–d) All graphs display means ± *SD*, significance from control denoted by asterisk; *p < .05, **p < .01. RT‐PCR, polymerase chain reaction

To examine the molecular mechanisms of regeneration, gene expression analysis was carried out on myogenic (Figure 4a), myosin heavy chains (Figure 4b), inflammatory genes (Figure 4c) and non‐myogenic developmental genes (Figure 4d). Myogenic markers Myod and Myog were expressed at significantly lower levels throughout regeneration (*p* = .013, *p* = .001), with the only myogenic marker expressed at an increased level being the embryonic myosin heavy chain, Myh3. Myh3 was significantly increased (*p* = .018) by 52% compared to control immediately following injury, however throughout regeneration transcription of this gene reduced across time to 45% of control at End DM 14 days later. Expression of myosin heavy chains associated with increased maturity, Myh8 (perinatal) and Myh1 (mature), initially were reduced (18% and 38%, respectively) followed by an increase through recovery from 4 days post injury onwards. At 14 days post injury (End DM), both Myh8 and Myh1 expression were elevated compared to control although not significantly.

Il6 was elevated 34‐fold in 2D experiments (Figure 1i), however was only up‐regulated 4.2‐fold in 3D (*p* = .024). Il6 expression returned to baseline 4 days (End GM) following injury and remained similar to control for the remainder of regeneration (Figure 4b). Mcp1 was elevated immediately following injury (fourfold, *p* = .011), and was expressed twofold higher than control at End GM time point although not significant. At the end of regeneration, Mcp1 expression returned to baseline levels (Figure 4b). To examine if injury was promoting non‐myogenic commitment of mononuclear cells, the markers Pparg (adipogenic) and Runx2 (osteogenic) were also quantified, although no significant variation from control expression was observed (Figure 4c).

To ensure that the addition of basement membrane components and growth factors contained within Matrigel® was not the sole cause of regeneration seen in tissue engineered constructs, further 2D experiments using Matrigel® coated coverslips were carried out. BaCl_2_ injury caused a significant reduction in fusion index (*p* < .001), that despite recovering significantly (*p* = .016), remained significantly (*p* < .001) below control at experimental termination (Figure S3). A proliferative phase was evident at End GM time‐point (Figure S3), in addition to consistent myotube widths pre and post injury. Significant reductions in nuclei per myotube were observed (*p* = .031); however, this was the only variation from the trends observed with gelatin as a substrate. Increased fusion index at control and following injury could potentially affect the regenerative process; however, correlation analysis shows no relationship between fusion, before or after injury, and the level of regeneration (Figure S4). This data confirmed the requirement for a 3D environment for full regeneration.

## 4 DISCUSSION

In this study, we present a model of skeletal muscle regeneration which requires no laboratory animal sacrifice or donor tissue and is designed to be straightforward to replicate between laboratories. In addition, we have made a direct comparison of monolayer to 3D culture to ensure that the increased complexity of 3D culture improves the biological relevance of results obtained.

In monolayer, C2C12 cultures showed limited regeneration following injury and so lacked a fully regenerative response. As the C2C12 cell line is a committed cell line, analysis of stem cell activation which is possible in primary derived myogenic precursors is not possible, however this cell line does allow the committed myogenic component of regeneration to be isolated. Therefore, the RNA levels of Myod and Myog have been examined to indicate the commitment of cells to myogenesis. An increase in Myod and Myog was observed only in the DM phase of recovery in monolayer cultures, suggesting a requirement for a change in serum concentration to trigger the myogenic program activating these canonical transcription factors. This recovery of Myod and Myog RNA levels but not of myotubes, suggesting a blockade of myogenesis at a late developmental stage. This blockade is most likely preventing the fusion of myoblasts into myotubes (Bentzinger, Wang, & Rudnicki, 2012). In addition to the potential myogenic blockade an increase in the non‐myogenic adipogenic transcription factor Pparg (Memon et al., 2000; Siersbæk, Nielsen, & Mandrup, 2010) suggest a proportion of cells in the culture commit to non‐myogenic developmental lineages, again blocking myogenesis. In contrast to monolayer cultures Myod and Myog were seen to fall continuously through recovery in engineered tissues, without the upregulation seen during the DM phase of regeneration in monolayer. As regeneration was morphologically complete by 4 days post injury, the upregulation of Myod and Myog required for myogenesis followed by subsequent down regulation (Bentzinger et al., 2012) may have occurred in the GM phase of recovery and therefore expression of these genes was resolved by 4 days post injury. Rapid induction of Myh3 (embryonic/regenerative) is seen immediately post injury, and is a response to injury consistent with *in vivo* models, which see preferential expression of regenerative/embryonic isoforms of myosin heavy chains during regeneration (Gorza, Sartore, Triban, & Schiaffino, 1983; Hindi & Kumar, 2016; Schiaffino, Rossi, Smerdu, Leinwand, & Reggiani, 2015). This response to switch to embryonic myosin may protect remaining myotubes from further injury and perhaps increase the ability of these myotubes to regenerate and support myogenesis. Throughout regeneration Myh3 expression falls and Myh8 (perinatal) and Myh1 (mature) isoforms increase in expression, showing a return to a mature phenotype and recovery from injury. At 14 days post injury increased Myh8 and Myh1 expression compared to controls suggests a more mature phenotype, and potentially explains the non‐significant increase in force production at End DM time point. Force generation in skeletal muscle is dependent upon a myriad of contributing factors and as such this is likely to be one of numerous contributing variables. Myh3 expression in monolayer matches the morphological trends, with no increased Myh3 expression immediately post injury. This suggest no isoform hand over as seen in engineered tissues, and instead an early blockade of myogenesis shown by the late elevation of Myod and Myog. This rapid regeneration of engineered tissues in GM highlights the regenerative capacity of the 3D environment, in the absence of external serum concentration changes. This inherent ability of the engineered tissues to produce the required cues to drive myogenesis without external signals is desirable, as muscle derived factors are known to drive regeneration *in vivo* and appear to be doing so in this engineered tissue.

Inflammatory factors present in regeneration have been shown to promote proliferation of myogenic cells (Hoene, Runge, Haring, Schleicher, & Weigert, 2013; Yahiaoui, Gvozdic, Danialou, Mack, & Petrof, 2008), and indeed a proliferative response post injury is observed in monolayer. When Il6 and Mcp1 expression were examined, only Il6 was upregulated immediately post injury and rapidly resolved. No induction of Mcp1 suggests an incomplete inflammatory response which may contribute to the reduced regenerative capacity of monolayer cultures. *In vivo* data suggests the major role of MCP‐1 is as a monocyte chemoattractant with its removal impairing regeneration (Shireman et al., 2006), and therefore the lack of Mcp1 expression reduces the biological relevance of the monolayer model. This limits its suitability for studying interactions with immune cells in future work narrowing the potential applications of monolayer studies. The lack of an improved regenerative response in the presence of basement membrane proteins, or increased culture time, suggest that monolayer cultures have an inherently limited regenerative capacity following a significant injurious insult.

Collagen I based tissue engineered skeletal muscles showed no regenerative capacity; however, the addition of Matrigel® was sufficient to allow regeneration following injury, showing the importance of basement membrane proteins in regenerative processes. Furthermore, the 3D organisation of these proteins must be a requirement for regeneration as basement membrane supplementation in monolayer was shown to be insufficient to support full regeneration. The presence of a niche with 3D organisation is key for defining stem cell proliferation, lineage commitment and self‐renewal in *in vivo* muscle and the 3D environment supplied by tissue engineered muscles is able to reproduce this in a way that is impossible in monolayer culture. The presence of this regenerative niche within collagen/Matrigel® engineered muscles allows potential future work to examine how different injury mechanisms, which may disrupt this niche, affect regeneration.

The full morphological regeneration of myotube number and full functional regeneration matches the response to BaCl_2_ insult in *in vivo* experiments (Hardy et al., 2016). The ability to measure functional output, something not possible in monolayer cultures, is a major advantage of 3D systems allowing direct and rapid assessment of tissue function which is the primary clinical measure of recovery from injury. The inflammatory response of 3D cultures was also shown to be more biomimetic than monolayer. Both Il6 and Mcp1 showed increased expression, compared to Il6 alone in monolayer, this increased number of inflammatory cytokines adds complexity to the regenerative environment post injury. As immune cells play an important role in directing and regulating skeletal muscle regeneration an inflammatory response as close to *in vivo* as possible will be required to understand the interaction between muscle and immune cells. As such we see the tissue engineered model presented as superior to the monolayer equivalent as it not only provides a fully regenerative response and functional output but expresses a fuller inflammatory response to injury important for the recruitment and activation of immune cells which support regeneration *in vivo*.

This study, which compares directly the use of the same injury mode in both monolayer and 3D cultures, demonstrates the usefulness of tissue engineered models for disease, and highlights where these models can be used to produce results superior to monolayer culture. We also recognise that monolayer culture is still widely used for preclinical interventions and have shown that a monolayer model of skeletal muscle regeneration is perhaps of use for some applications which require only limited biological relevance. Previous work demonstrating regeneration in tissue engineered muscles relies upon isolation of myogenic precursors from rat tissue (Juhas et al., 2014, 2018), whereas this study presents a cell line based model contained within a 3D printed mould making it suitable from high throughput work often required in therapeutic development. We show that regeneration of tissue engineered skeletal muscles containing basement membrane proteins provides a system which is representative of the *in vivo* response to chemical injury, providing a platform for the investigation of the myogenic events which underpin regeneration. This model can be used as a tool for researchers to obtain *in vivo* like results without requiring laboratory animals, improving the relevance of preclinical screening and potentially reducing failure rates of novel clinical interventions.

## CONFLICT OF INTEREST

The authors have no conflict of interest to declare.

## AUTHOR CONTRIBUTIONS

J.F. contributed to the design of experiments, performed all experimental work and data analysis and drafted the manuscript. A.C., R.R., and D.P. contributed significantly to experimental design and critically reviewed the manuscript throughout the drafting process. A.S. contributed to experimental design. M.L. conceived the concept of the work, contributed to experimental design and reviewed the manuscript.

## Supporting information


**Figure S1 Dose response and time course of BaCl**_**2**_ **in monolayer cultures. (a)** 10x phase contrast images showing removal of myotubes, which appear as bright linear structures, from differentiated C2C12 cultures. **(b)** 10x phase contrast images of fine dose screen. Scale bar represents 100 μm

**Figure S2 Extended recovery does not rescue reduced fusion index following BaCl**_**2**_ **insult. (a)** 20x fluorescence micrographs along an extended recovery time course. Stained with phalloidin (red) and DAPI (blue), Scale bar 50 μm **(b)** Fusion index of cultures. Mean ± SD **(c)** Total nuclei per image frame. Mean ± SD. Asterisks above bars denote significance from control at a level of *p* < 0.001

**Figure S3 Basement membrane proteins alone cannot support regenerative recovery. (a)** 20x micrographs stained for actin (Rhodamine, Red) and nuclei (DAPI, Blue). Scale bar 50 μm. **(b)** Fusion index **(c)** Nuclei per image frame **(d)** Myotube width (μm) **(e)** Nuelci per myotube **(b‐e)** All graphs display mean ± SD, significance from control * *p* < 0.05, ** *p* < 0.01, *** *p* < 0.001.

**Figure S4 Correlation of fusion index with regeneration in monolayer cultures (a)** Fusion index at control correlated with fusion at End DM divided by fusion at control defined as regenerative index. **(b)** Fusion index at 0 Hrs correlated with regenerative index.

**Figure S5 Characterisation of tissue engineered muscle (a)** Cross section image of collagen/Matrigel® C2C12 hydrogel. Green – MyHC, Blue – Nuclei, Scale bar 100 μm **(b)** Macroscopic image of collagen/Matrigel® hydrogel showing the extent of hydrogel deformation. Scale bar 3 mm
