# Supplementary figures and images for: Functional regeneration of tissue engineered skeletal muscle in vitro is dependent on the inclusion of basement membrane proteins
Source: Cytoskeleton (Hoboken). 2019 Aug 19;76(6):371–82. doi: 10.1002/cm.21553 (PMC6790946; doi:10.1002/cm.21553)

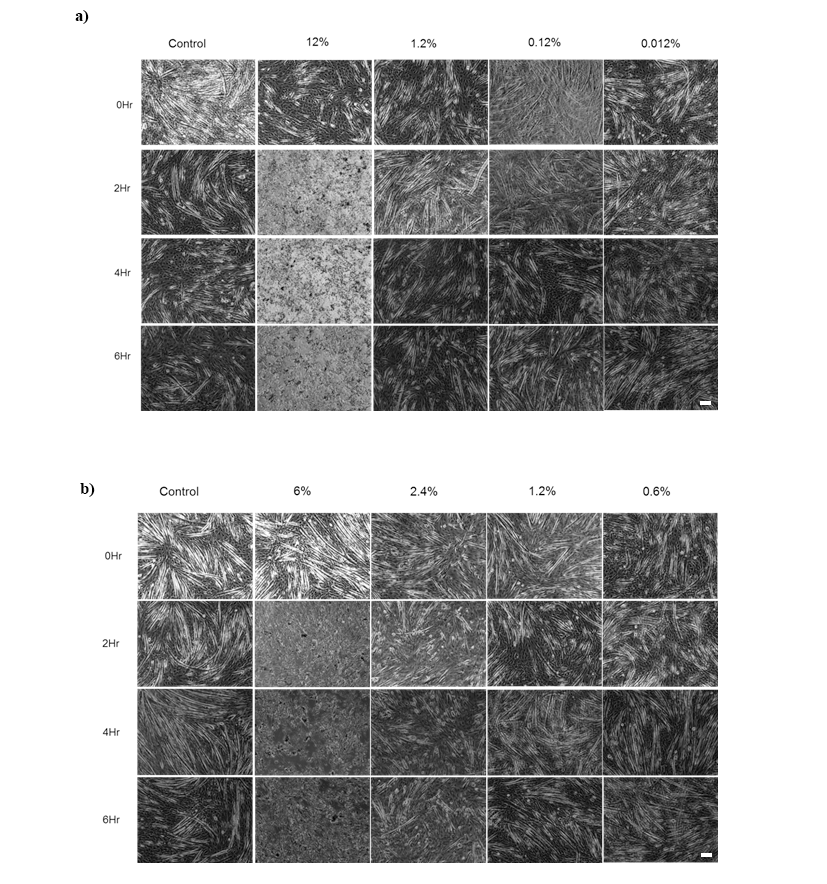

Supplement: Supplementary file 1 — Figure S1 Dose response and time course of BaCl 2 in monolayer cultures. (a) 10x phase contrast images showing removal of myotubes, which appear as bright linear structures, from differentiated C2C12 cultures. (b) 10x phase contrast images of fine dose screen. Scale bar represents 100 μm [file CM-76-371-s001.tif]

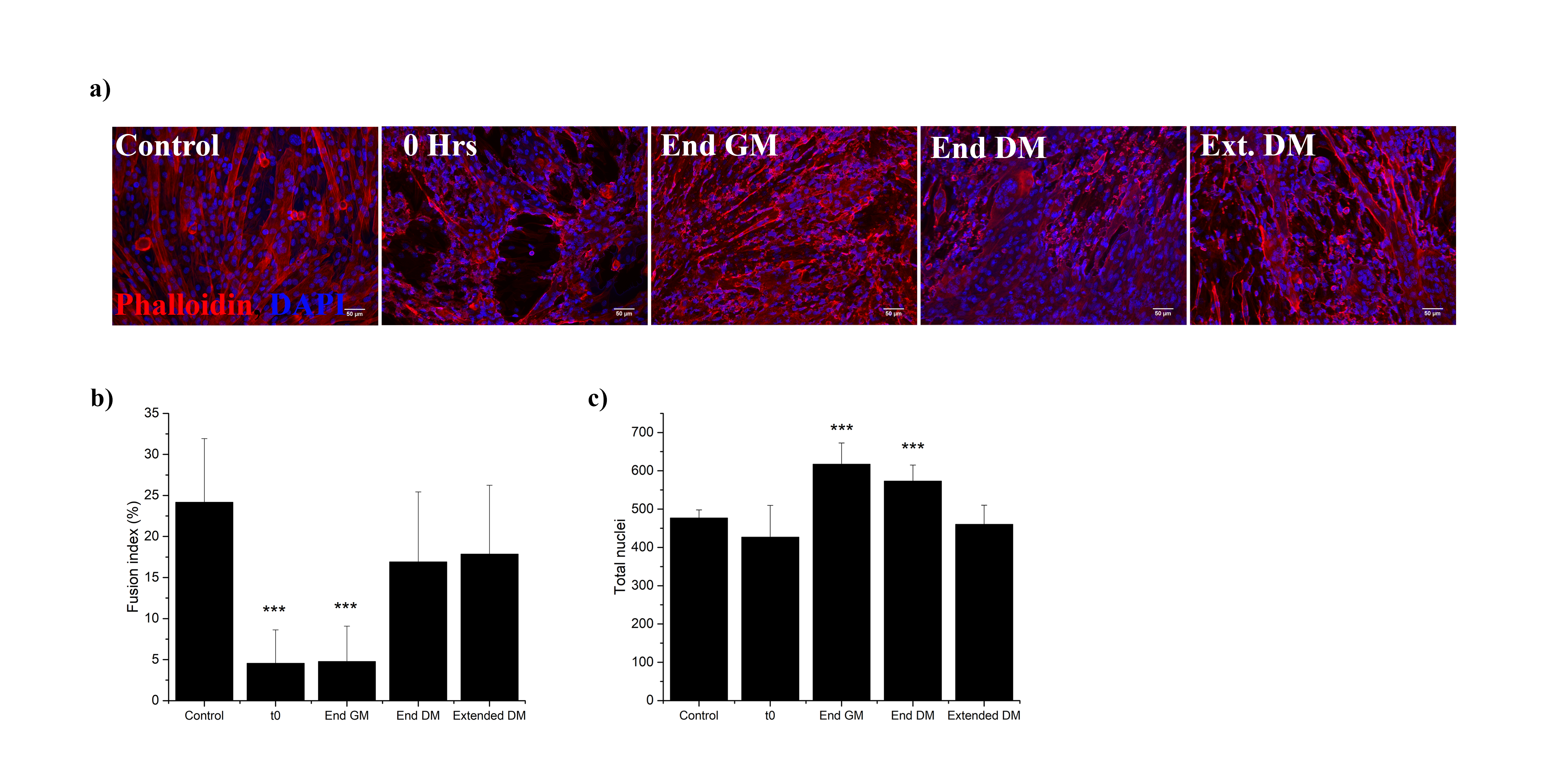

Supplement: Supplementary file 2 — Figure S2 Extended recovery does not rescue reduced fusion index following BaCl 2 insult. (a) 20x fluorescence micrographs along an extended recovery time course. Stained with phalloidin (red) and DAPI (blue), Scale bar 50 μm (b) Fusion index of cultures. Mean ± SD (c) Total nuclei per image frame. Mean ± SD. Asterisks above bars denote significance from control at a level of p < 0.001 [file CM-76-371-s002.tif]

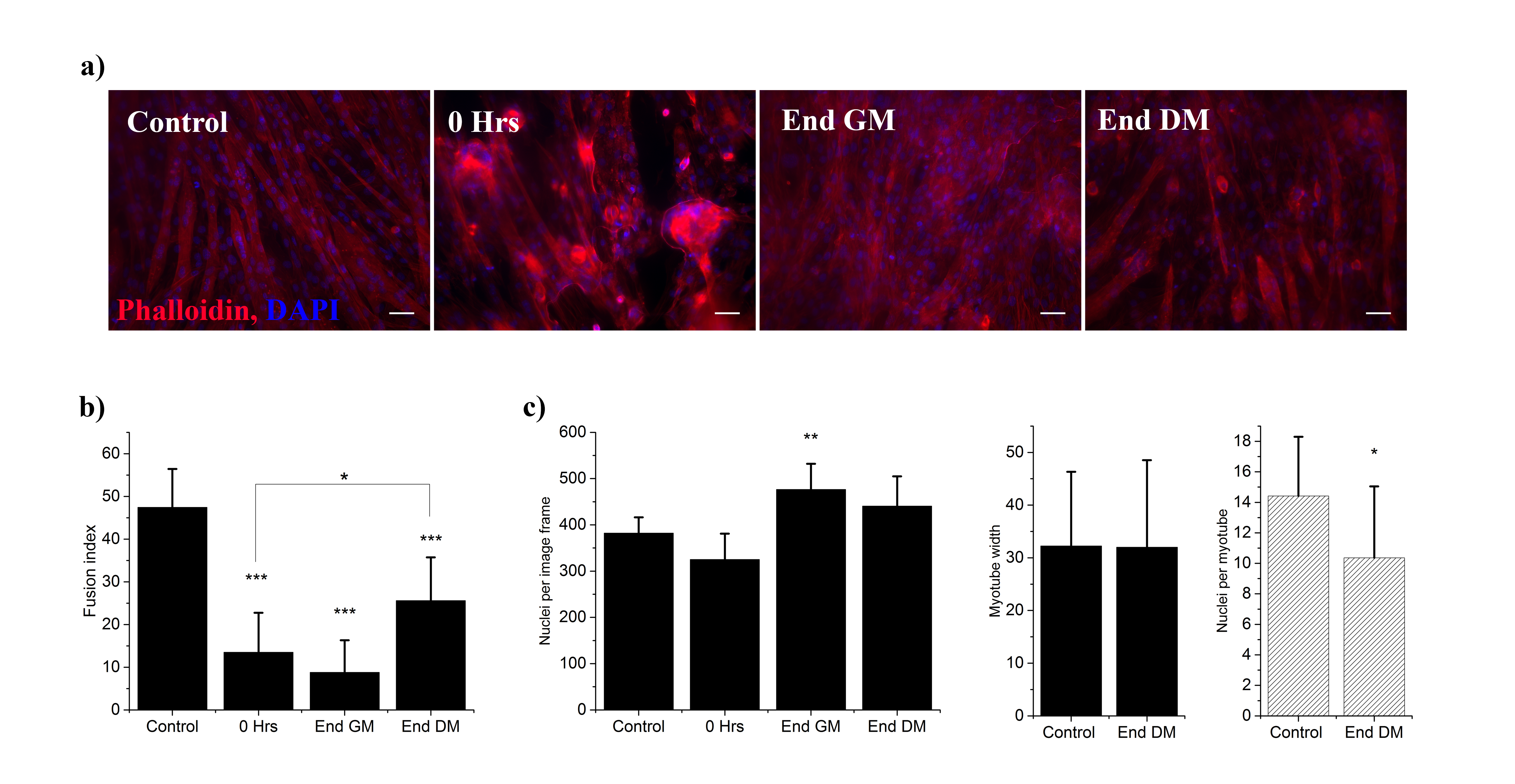

Supplement: Supplementary file 3 — Figure S3 Basement membrane proteins alone cannot support regenerative recovery. (a) 20x micrographs stained for actin (Rhodamine, Red) and nuclei (DAPI, Blue). Scale bar 50 μm. (b) Fusion index (c) Nuclei per image frame (d) Myotube width (μm) (e) Nuelci per myotube (b‐e) All graphs display mean ± SD, significance from control * p < 0.05, ** p < 0.01, *** p < 0.001. [file CM-76-371-s003.tif]

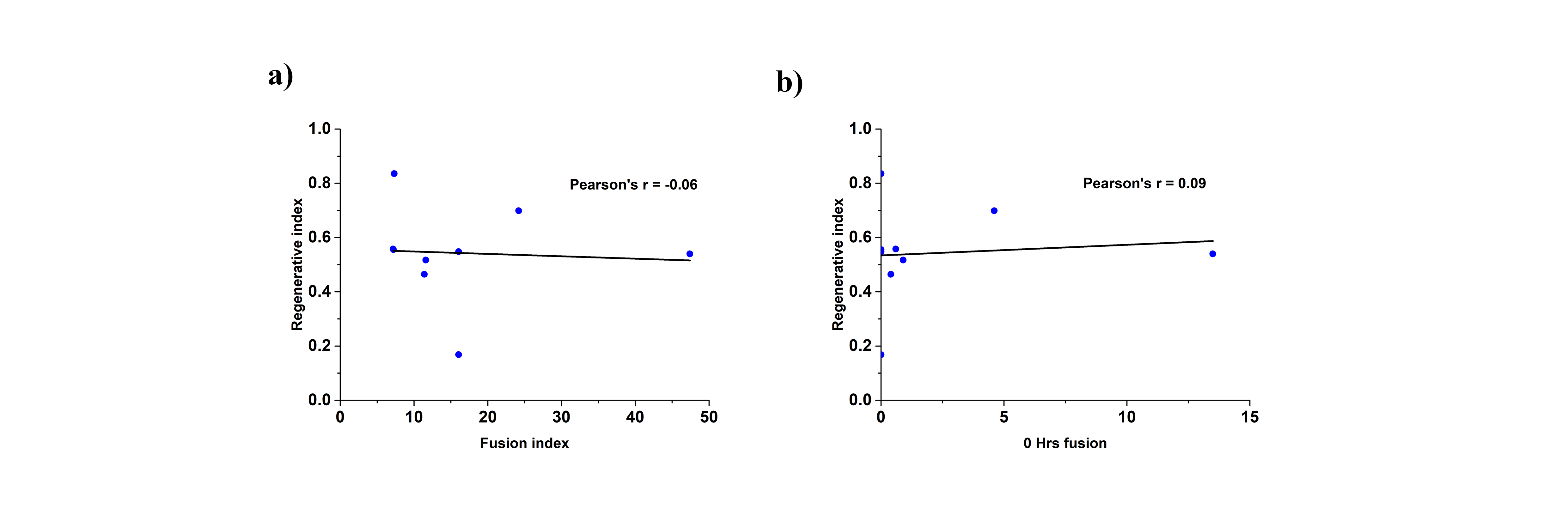

Supplement: Supplementary file 4 — Figure S4 Correlation of fusion index with regeneration in monolayer cultures (a) Fusion index at control correlated with fusion at End DM divided by fusion at control defined as regenerative index. (b) Fusion index at 0 Hrs correlated with regenerative index. [file CM-76-371-s004.tif]

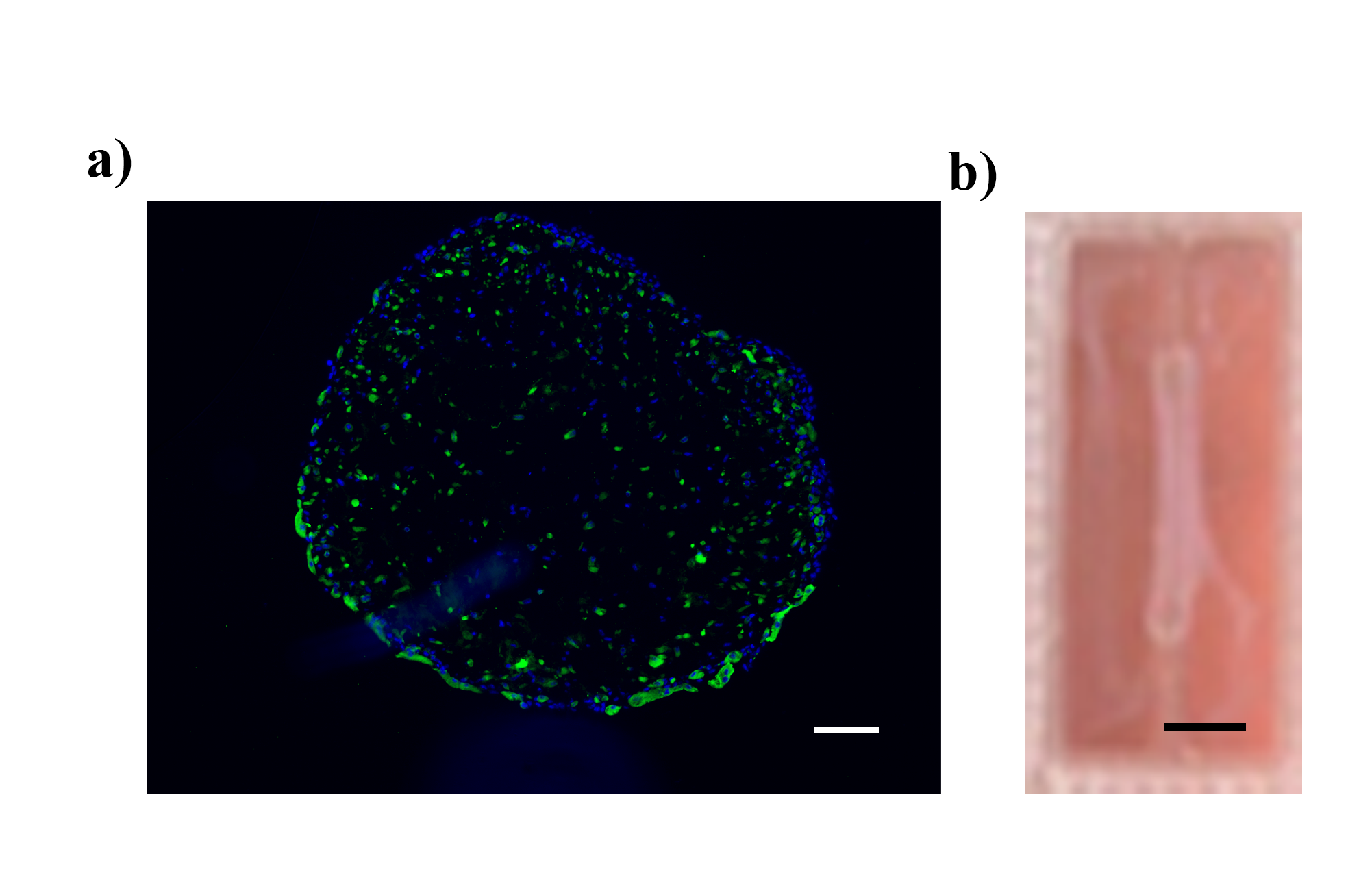

Supplement: Supplementary file 5 — Figure S5 Characterisation of tissue engineered muscle (a) Cross section image of collagen/Matrigel® C2C12 hydrogel. Green – MyHC, Blue – Nuclei, Scale bar 100 μm (b) Macroscopic image of collagen/Matrigel® hydrogel showing the extent of hydrogel deformation. Scale bar 3 mm [file CM-76-371-s005.tif]
